# An online tool for obesity intervention and public health

**DOI:** 10.1186/s12889-016-2797-3

**Published:** 2016-02-10

**Authors:** Jason G. Su

**Affiliations:** Environmental Health Sciences, School of Public Health, University of California at Berkeley, Berkeley, CA 94720-7360 USA

## Abstract

**Background:**

Though the United States of America (U.S.A.) obesity rate shows signs of leveling off, rates remain high. Poor nutrition contributes to the development of obesity, and physical inactivity is an important cause of numerous diseases and directly linked to obesity. Efforts to improve diet, increase physical activity and pursue other behavioral changes seem imperative. However, the effective management of intervention strategies for large number of participants are challenging because services in primary, secondary, and tertiary cares are often under-resourced, relatively uncoordinated with other parts of the health system. It is thus necessary to have accompanying intervention strategies that can be carried out at population level. In this paper, we describe an online intervention tool designed for the Obesity Prevention Tailored for Health II project to help achieve such goals.

**Results:**

The first part of the online tool locates healthy food stores and recreational programs within a specified distance of a participant’s home or a place of interest. The food environments include fruit & vegetable stores, farmers’ markets and grocery stores, and the companying popup window shows the street address and contact information of each store. The parks and recreational programs are displayed on names of park or recreational program, types of program available, and city each amenity belongs to. The tool also provides spatial coverage of vegetation greenness, air pollution and of historical traffic accidents involving active travel.

The second part of the tool provides optimized travel options for reaching various amenities. By incorporating bicycling, walking and public transit into the trip planner, this online tool helps increase active transport and reduce dependence on automobiles. It promotes transportation that encourages safety awareness, physical activity, health, recreation, and resource conservation.

**Conclusions:**

We developed the first Google-based online intervention tool that assists obese and overweight participants in finding food and recreational amenities around locations of interest and identifying optimized routes that fit their personal preferences. This tool can also serve general public and policy makers for education, disease prevention and health promotion.

## Background

During the past 20 years, there has been a dramatic increase in obesity in the United States (U.S.) and rates remain high. More than one-third of U.S. adults (34.9 %) and approximately 17 % (or 12.7 million) of children and adolescents aged 2–19 years are obese [1–3]. Overweight and obesity have serious immediate health consequences for both the individual and the broader community [4–6]. A wide range of evidence from epidemiology, case–control studies, and clinical trials have identified poor nutrition intakes, such as increased intakes of energy dense nutrient foods and sweetened drinks, low dietary fiber intakes, and large portion sizes, contribute to the development of obesity [7, 8].

Though obesity is associated with various chronic diseases and even early death, physical inactivity is an important cause of numerous diseases and directly linked to obesity [9, 10]. The U.S. Centers for Disease Control and Prevention (CDC) recommended amounts of physical activity to confer important health benefits: 150 min of moderate-intensity aerobic activity (i.e., brisk walking) every week and weight training muscle-strengthening activities on 2 or more days a week [11].

Although the evidence on what is effective in treating obesity is still emerging [12–17], efforts to improve physical activity in addition to diet and other behavioral changes seem imperative. In the Obesity Prevention Tailored (OPT) for Health II project [18], intervention strategies were designed to limit growth in Body Mass Index (BMI), increase the consumption of fruit and vegetables, reduce the consumption of saturated fat, increase moderate/vigorous physical activity, and reduce sedentary through interview, in-person meeting and tailored print materials. The effective management of these interventions for a small number of participants is feasible; however, this management is ineffective for large number of participants because services in primary, secondary, and tertiary cares are often under-resourced, relatively uncoordinated with other parts of the health system [8]. Given the increasing popularity of Internet use, its low cost, adaptability, and scalability for intervention strategies, we developed the first online obesity intervention tool to facilitate implementing the OPT II project. The intervention tool was developed through a familiar and user-friendly Google Maps interface for use by participants served by the two Medical Centers in the Los Angeles Metropolitan Area. The online intervention tool was aimed at helping participants interactively locate available healthy food stores around their neighborhoods to discover new dietary options, find available parks and recreational programs for possible increase in physical activity, and identify optimized transportation methods.

## Implementation

The OPT intervention study was designed and conducted by investigators from Claremont Graduate University, Kaiser Permanente Southern California (KPSC), the University of Southern California, and the University of Colorado at Denver. The protocol was reviewed and approved by the respective institutional review boards (IRBs) for protection of the human subjects. The IRBs approved the study included Claremont Graduate University, Kaiser Permanente Southern California (KPSC), the University of Southern California, and the University of Colorado at Denver. The goal of the study was to test the effectiveness of a family-based intervention targeting four behaviors: increased consumption of fruits and vegetables; decreased consumption of saturated fat; increased physical activity; and decreased sedentary time. The OPT intervention was delivered to a parent and his/her 10- to 12-year-old child. To be eligible, the parent had to be a member of the KPSC health plan for at least 1 year and both the parent and child had to be an English speaker. The KPSC interviewer requested permission to disclose the name and contact information of the parent to Claremont Graduate University research staff when eligible parents expressed interest in the study. These families were labeled as “acceptors” and parents and their children were scheduled for a 2-hour in-person appointment to obtain written informed consent from parents and assents from children. The detailed design of the study could be found from Ghai et al. [18]. This paper focuses on the online intervention part of the project.

**Fig. 1:**
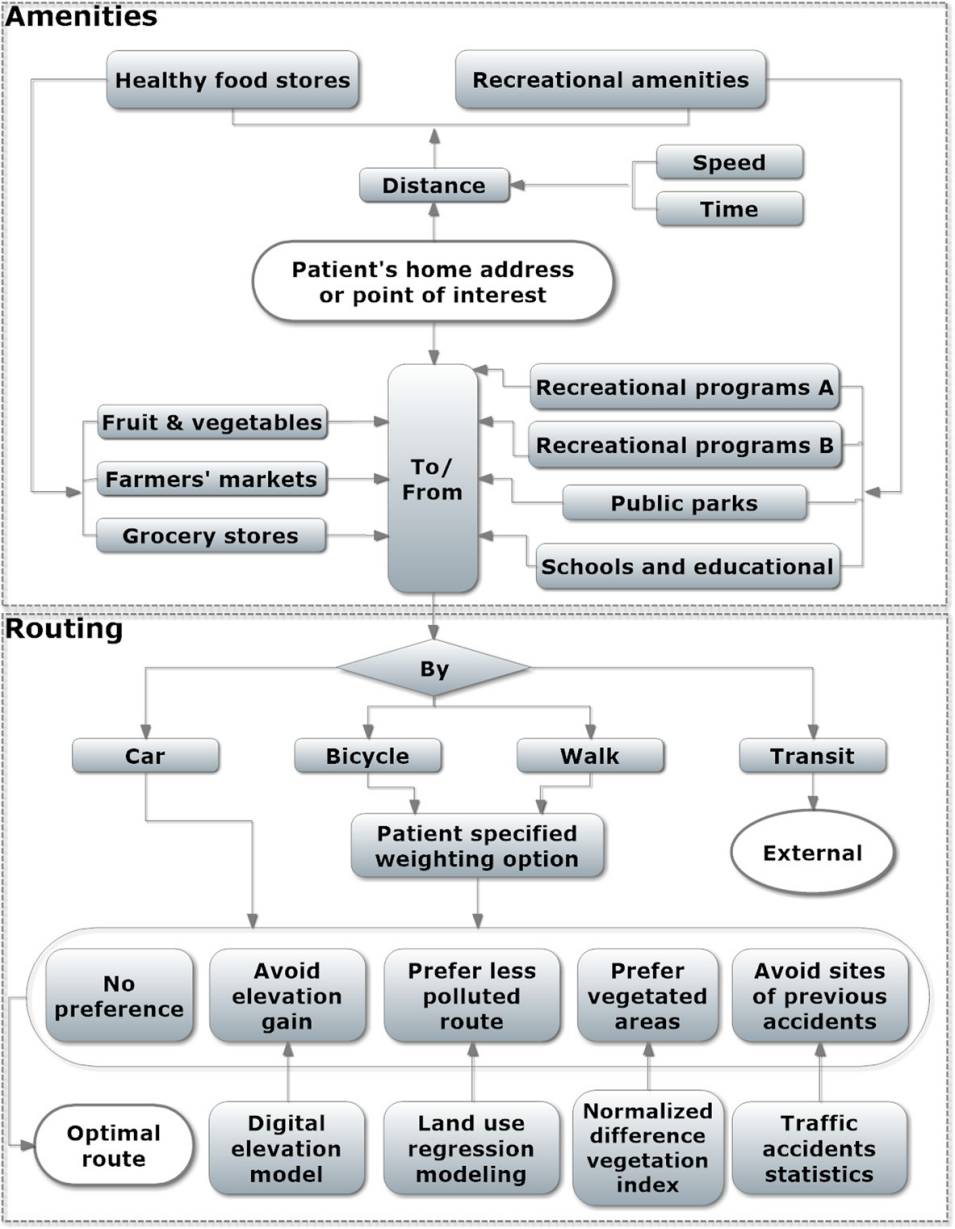
The functionality of the Google-based online intervention tool with the top part representing the amenities of the system and the bottom part the optimal routing option to get to or come back from those amenities

The main functions of the Google-based online intervention tool are shown in Fig. 1. The top part helps a participant locate healthy food stores and recreational programs within a specified buffer distance of the participant’ home or place of interest. The bottom part identifies optimized travel options to reach an amenity including active travel through bicycling and walking. The following sections detail criteria for selecting amenities that might help prevent overweight and obesity, on how the amenities are incorporated into the system, and on the trip planning algorithms used to help participants participate in active transport for reaching their amenities.

### Healthy food amenities

**Fig. 2:**
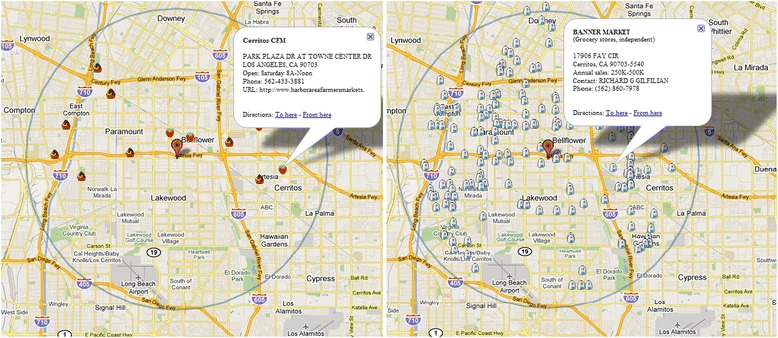
Left: Fruit & vegetable stores (marked using *fruit basket icons*) and farmers’ markets (*strawberry icons*) within 5 miles of the Kaiser Permanente Bellflower Medical Center in Los Angeles. Right: Grocery stores (*milk bottle icons*) within 5 miles of the same location

Fruit and vegetables have a low energy density because of their high content of water, low content of energy [19] and they have high content of dietary fiber, which is considered to increase satiety and reduce feelings of hunger [20]. Fruit and vegetables also contain flavonoids, a group of nonnutritive phytochemicals that may have antiobesity effects [21, 22]. High fruit and vegetable intakes were shown in some studies inversely associated with weight gain [22] and might lead to eating fewer high-fat foods [23]. To serve the purpose of possible weight gain intervention, we geocoded fruit & vegetable stores and farmers’ markets for the Los Angeles Metropolitan Area, including the Los Angeles County, western part of the San Bernardino County and the northern part of the Orange County. Data were acquired from the Environmental Systems Research Institute (ESRI, Redlands, CA) and the California Department of Public Health. Fruit basket markers are used to represent fruit & vegetable stores and each popup window shows the street address and contact information of a store (Fig. 2, left). For farmers’ markets (strawberry markers in Fig. 2), additional information includes time of opening and if available a link to the website of a farmers’ market. Figure 2 on the left shows the locations of fruit & vegetable stores and farmers’ markets within 5 miles of the Kaiser Permanente Bellflower Medical Center. In addition to fruit & vegetation stores and farmers’ markets, grocery stores including supermarkets are also considered as important contributors to nutrition among residents of neighborhoods [24]. We also included grocery stores in the region (Fig. 2 right) for the online obesity intervention program, including convenient stores, cooperative food stores, drug stores, grocery stores, supermarkets and warehouse club stores with annual sales ranging from less than $50,000 to over 1 million dollars. Similar to fruit & vegetable stores and farmers’ markets, a grocery popup window includes its street address and contact information. To show on a map the healthy food stores within a distance of location of interest, data are extracted from an Access database using Asynchronous JavaScript and eXtensible Markup Language (AJAX) techniques and web services in C#.

### Parks and recreational amenities

One way to help address the epidemic of obesity in the United States is an ambitious parks and recreation program [25]. Parks and green spaces provide many environmental, social and psychological services that are of significance for the livability of modern cities and the wellbeing of urban dwellers [26, 27]. In addition, parks and green space often serve as sites of physical activity and may be an effective health promotion target for increasing active living in youth [28]. Wolch et al. [29] found that young children with better access to public recreational programs are less likely to become obese. Though some studies showed that reducing dietary energy density or increasing physical activity alone did not result in a weight loss or obesity rates [30–33], research found a significant greater survival rate for breast cancer participants who followed a healthy lifestyle that included both recommended intakes of vegetables-fruits and moderate levels of physical activity [34].

**Fig. 3:**
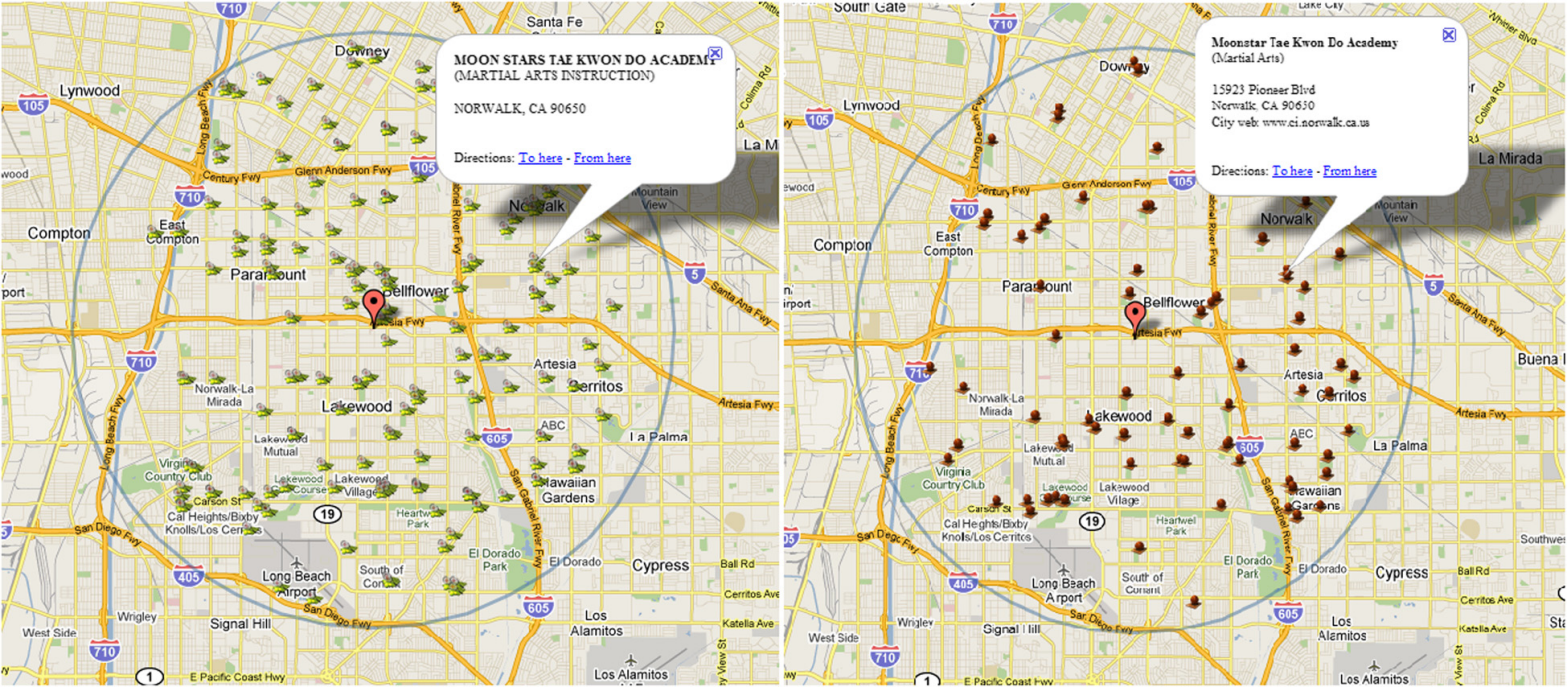
Left: Recreational programs derived from ESRI business analysis (marked using *tennis racket and court icons*) within 5 miles of the Kaiser Permanente Bellflower Medical Center in Los Angeles. Right: Recreational programs based on detailed local audit of related resources (*basketball and court icons*) within 5 miles of the same location

The strong protective effect of interaction led us to test if in addition to dietary interventions, integrating public parks and recreational services at the community neighborhoods would help prevent childhood obesity. The public parks used in this study were created by pooling together data from the following sources: ESRI’s Business Analyst, land use/land cover data from the Southern California Association of Governments (SCAG, Los Angeles, CA), coastal access information from the California Coastal Commission (Long Beach, CA), and Thomas Brothers Maps (Rand McNally, Irvine, CA), with the latter used mainly for cross-referencing and verification. Recreational programs included both general and detailed categories. The programs in the general category was derived from the ESRI’s Business Analyst and it includes the name of a recreational program, the types of programs available, and the city it belongs to (Fig. 3 left). Detailed recreational programming data (Fig. 3 right) were collected by means of an audit of public recreation courses performed during the summer 2006. Recreational offerings were gathered from municipal park and recreation brochures and materials. Most data were collected via internet search (though some direct telephone contact/mailings were necessary). Each listing for sports, fitness, or other recreational programs that required a metabolic equivalent (MET) value greater than 3.0 (physical exertion equivalent to moderate walking) was included in the database. All courses had to be sponsored by the city, but could occur at either a city park or community recreation center, or a non-city owned site. Addresses were geocoded in ESRI ArcView 9.2 and verified with Teleatlas.

A participant has options to select amenities of specified distance around his/her home or at a place of his/her interest as shown in Figs. 2 and 3. A participant can obtain detailed information for each amenity by left-clicking the amenity for a popup window.

### Vegetation greenness and pollution surface

**Fig. 4:**
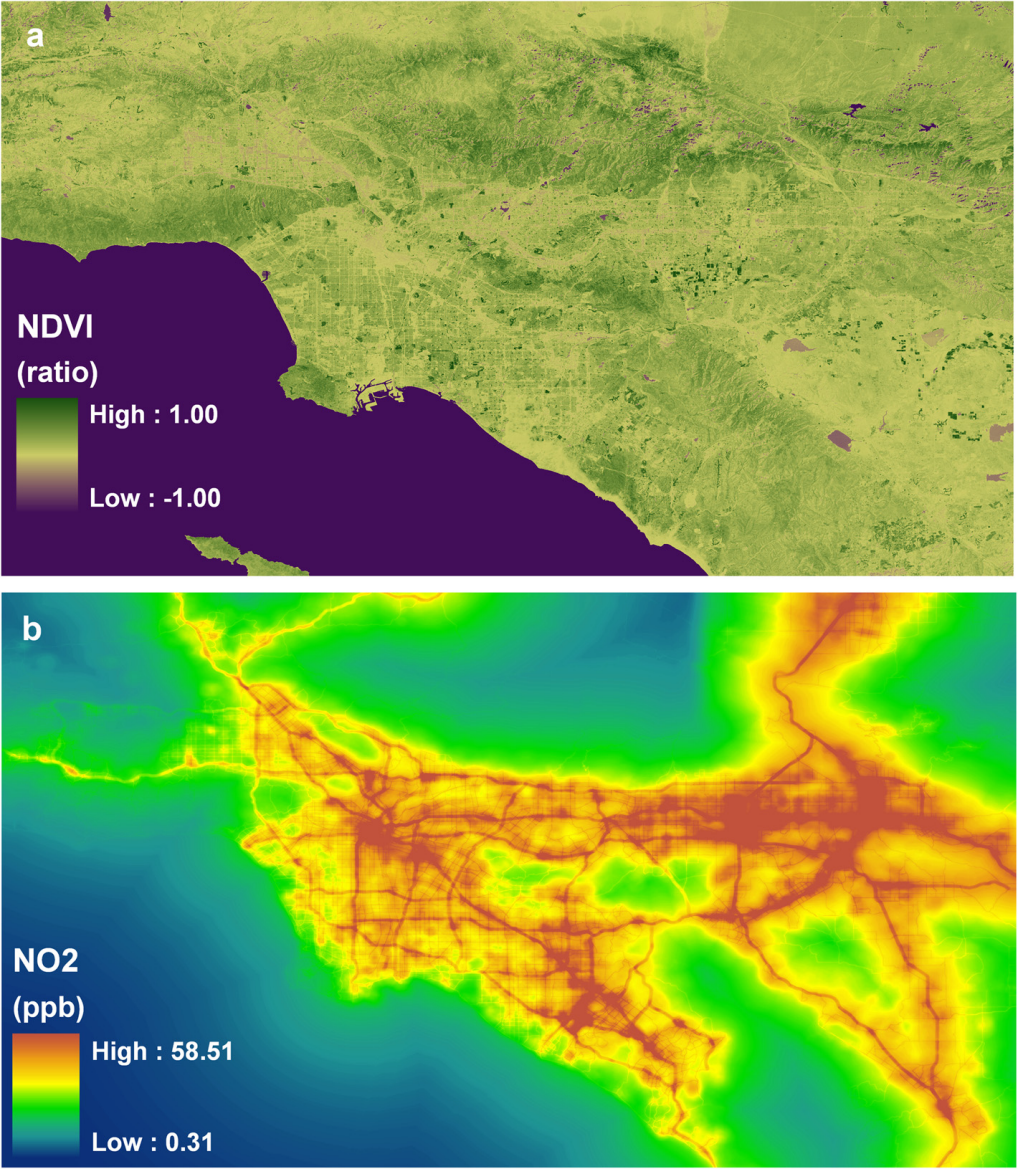
Vegetation greenness through NDVI (normalized difference vegetation index) (**a**) and predicted surface of NO_2_ from traffic-related air pollution (**b**)

**Fig. 5:**
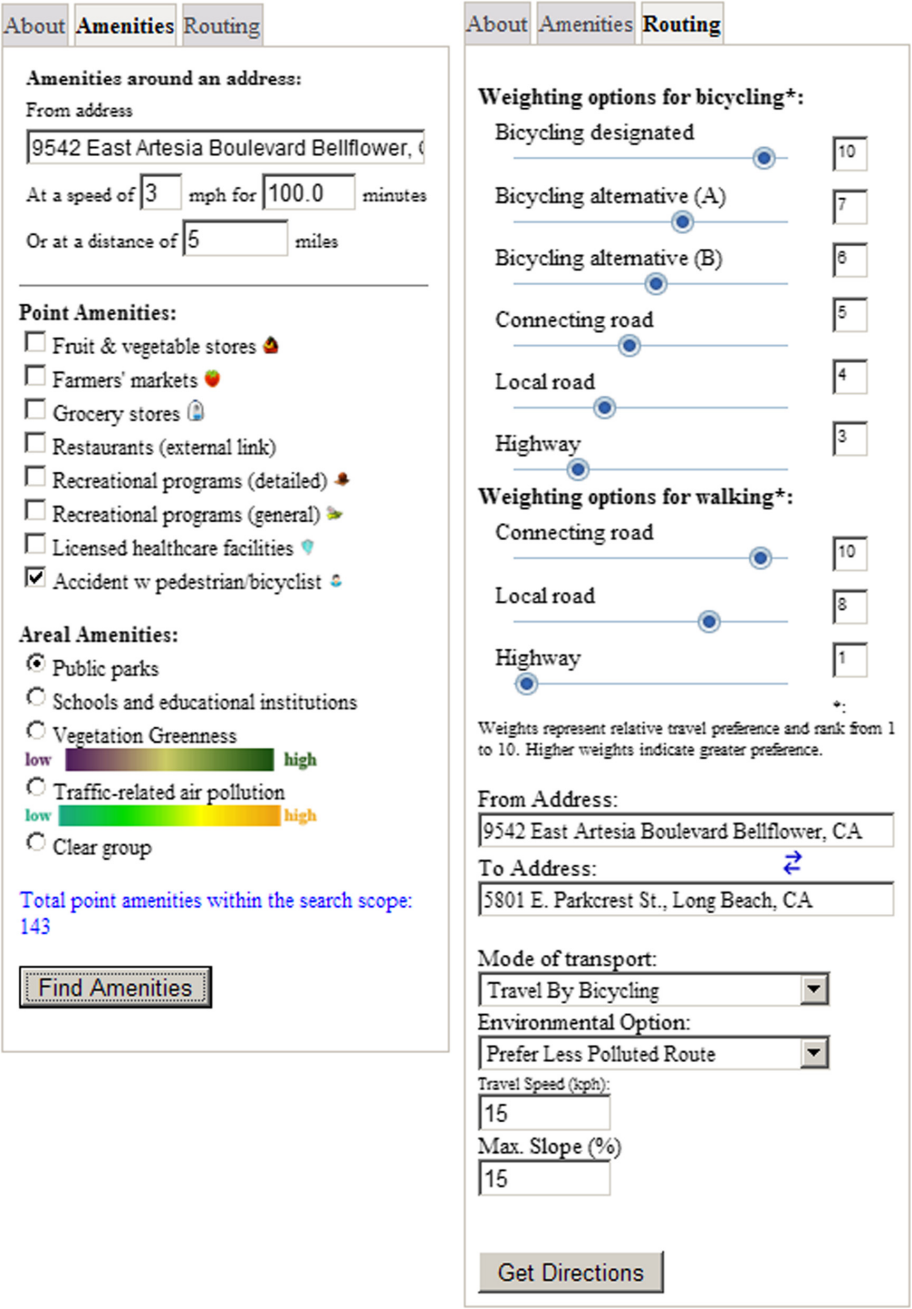
Amenities (*left*) and routing (*right*) tabs for the Google-based online intervention tool

Environmental changes and heat waves have significant effects on plants, communities and ecosystems including human being [35, 36]. At the same time, the presence of vegetation provides shading and converts incident solar radiation to latent heat [37, 38]. Normalized difference vegetation greenness (NDVI) is used to represent vegetation greenness. Live green plants absorb solar radiation in the photosynthetically active radiation (PAR) spectral region and scatter (i.e., reflect and transmit) solar radiation in the near-infrared spectral region. Live green plants appear relatively dark in the PAR and relatively bright in the near-infrared. The NDVI is calculated as follows:

$$ NDVI = \frac{\left(NIR- RED\right)}{\left(NIR+ RED\right)} $$

where RED and NIR stand for the spectral reflectance measurements acquired in the red and near-infrared regions, respectively. These spectral reflectance take on values between 0.0 and 1.0 and NDVI itself varies between −1.0 and +1.0. A higher NDVI index represents a greater vegetation greenness. The NIR and RED data for the study region were acquired from the US Geological Survey Landsat Enhanced Thematic Mapper Plus (ETM+) for 2001. Derived NDVI surface (Fig. 4a) were re-coded from low to high on the online intervention legend (Fig. 5a).

Increasingly, air pollution is found associated with disease and mortality [39–42]. Evidence is starting to show that air pollution exaggerates adipose inflammation, insulin resistance and other symptoms to people with diabetes, obesity and hypertension [43–46]. To help participants aware of the levels of air pollution in areas of their interest and if possible to avoid high polluted areas, we modeled traffic related air pollution (the main source of air pollution in the region) for the study region (Fig. 4b) using a land use regression modeling strategy [47]. The land use regression method seeks to predict pollution concentrations at a given site based on surrounding land use, road network, traffic, physical environment and population characteristics using a series of buffers [48]. Similar to vegetation greenness, we recoded the pollution values from low to high on the online intervention legend (Fig. 5a).

To speed up the display of the vegetation greenness surface and the air pollution surface, each surface is dissected into 80 rectangular segments. Each time only the selected image that intersects the scope of display will be called from the Access database and be overlaid on top of the current map. For safety awareness, we also incorporated traffic accident data that involved at least a bicyclist or a pedestrian from year 2005 to 2007. The data were acquired from the Statewide Integrated Traffic Records System (SWITRS) by the California Highway Patrol. Data were gecoded and stored in an Access database. Similar to the healthy food amenities, AJAX and web services were used to display locations and counts of accidents on the Google map. Other amenities selected for the online intervention tool included locations of restaurants (external link), schools and educational institutions, and licensed healthcare facilities (Fig. 5a).

To enable a layer to be displayed on the Google map, a user needs to select types of amenity and then click the “Update Selection” button. Because of overlapping issues associated with public parks, schools and educational institutions, vegetation greenness and traffic-related air pollution, only one of the four layers was allowed to be shown at a time. A user can also check with amenities within a specified walking, bicycling or driving distance by specifying travel speed and time spent in travel or simply by specifying a distance and then click the “Search Amenities Within” button.

### Optimized trip planner

The findings of the Cycling in Cities survey [49] and the growing number of general online route planners (e.g., Google Maps, Google Transit, Microsoft Live Search, MapQuest and Yahoo Maps) suggest an increasing demand for spatial decision tools for everyday transport. Some research [49, 50] suggests that bicyclists would use bicycles for more trips if they had trip information ahead of time. The Google-based bicycling trip planner developed for Metro Vancouver [51] allows individuals to choose routes based on user-stated preferences regarding safety, distance, elevation gain, air quality, and areas featuring trees and other vegetation. The trip planning tool for the OPT Health II is a tailored and enhanced tool that includes features not only identifying locations of general public’s interests, but also seeking locations of the healthy food stores and recreational program amenities in the region. To assist a participant in getting to an amenity or go to place after visiting an amenity, the “To Here” and “From Here” options are provided on the amenities popup windows (Figs. 2 and 3). To help participants and their communities achieve a significant shift from environmentally harmful and sedentary travel to environmentally friendly and active travel, this intervention tool also advocates for transportation that encourages and promotes safety, physical activity, health, recreation, and resource conservation. The optimized trip planner includes options not only for bicycling, but also for walking and public transit for the purpose of increasing active transport and reducing dependence on automobiles. In case a participant can only drive by car from or to an amenity, car driving is also provided as an option. To promote travel safety, traffic accidents involving at least a bicyclist or a pedestrian are included in the trip planning options. To maximize a participant’s participation in active transport, the tool also provides flexible weighting options for walking and bicycling. A participant can weight a bicycling choice using a scale from 1 to 10 for roadways to ride on, including bicycling designated, bicycling alternative, connecting roadway, local road and highways. For walking, the weighing include options for connecting road, local road and highways, also at a scale 1–10.

The details of the trip planning component are shown in Fig. 5b. In summary, there are four modes of transport: travel by car driving, bicycling, walking and public transit. For bicycling, a participant can further select using either the most bicycle friendly lanes or balanced routes. The most bicycle friendly option put those designated and high quality bicycle roadways at a higher priority compared to the balanced option. A participant can also specify travel speed and limit maximum slope gradient for both bicycling and walking options. The slope gradient is defined as the ratio of the altitude change to the horizontal distance between any two intersections of a road segment. When the ratio equals to 1 (i.e. 100 %), the slope in degree is 45°. Under each mode of transport, a participant can also select environmental preference for a route, including no restriction, avoid elevation gain, prefer low traffic pollution, prefer vegetated route and avoid sites of previous traffic accidents. Elevation gain is the total elevation increased during the whole trip. Traffic accidents are the total number of accidents with at least a pedestrian or a bicyclist during the last 3 years (2005–2007) on a chosen trip. No route length is taken into consideration for elevation gain and traffic accident preferences. For traffic pollution and vegetation greenness preferences; however, road length is considered. For example, total traffic-related air pollution is calculated based on the possible total inhalation on a chosen route, i.e. pollution = sum(road length_i_ * mean pollution on road segment_i_). Accidents involving a pedestrian or a bicyclist are provided only as a guidance for bicycling and walking safety.

For the trip planner component, asynchronous web services were developed using C#. One of the oldest and most widely used approaches in network optimization is the shortest path analysis (SPA) [52–54]. In our application, we are interested in searching for the shortest path with cost impedances for attributes based on the user-defined preferences. Many optimization problems are solved using a matrix of dimension n by n (e.g., [52] by various constraints [55–57]). These algorithms usually search more neighboring nodes or roads than those physically adjacent to the node of interest. An alternative to SPA that saves processing time is the use of topology. Since networks usually have a topological structure that identifies how the various features relate to one another (e.g., position, orientation, adjacency and connectivity), Su et al. [51] used the topological relationship of nodes and roadways and efficiently stored them in an index table and loaded them into memory when a client launches the bicycling trip planner. This works effectively (3–4 s to calculate a route) for the Greater Vancouver area in which 66,000 vertices (used for color-coded route output) and 22,500 roadway segments were used for the analysis; however, when applied to the Los Angeles Metropolitan Area, the index table was seen very inefficient. It took about 3 min to get a route for the 648,510 roadways and 2,025,053 vertices. To speed up time used for trip planning, a rectangular box with a half mile buffer distance around the start and destination addresses a participant specified is used for initiating the routing process. This algorithm significantly reduces the number of roadways needed in the computing process and thus significantly reduced the time required for calculating a route. Up to date, almost all the routing algorithms including those topology-based ones use nodes (i.e., road intersections) to guide a route selection process. The disadvantage of this method is its reliance on two separate tables: nodes and links (i.e., roadways) [51]. We further simplified the algorithm by using links as the basic selection process. In simple terms, the topology-based optimal route algorithm first identifies the starting roadway of a trip and marks it. All the physically adjacent roadways of unmarked within the buffered rectangular box a participant can travel to are then marked, and their impedances calculated and added to the corresponding total impedance. The marked roadway with the least total impedance to the original starting roadway is identified and used as a new starting roadway to find its physically adjacent and unmarked travelable roadways within the rectangular box. This process continues until the newly specified start roadway meets the destination roadway (i.e., a route is found) or until the two never merge after assessing all the travelable roadways within the rectangular box (no direct route). The link based algorithm does not require the usage of nodes and thus significantly improves the performance of the trip planner.

In the trip planner output window, carbon dioxide (CO_2_) emissions from driving by car and “savings” from cycling and walking are calculated and displayed. We assume that a typical passenger car emits 5 tons of CO_2_ from an annual travel distance of 20,000 km, which is equivalent to 0.25 kg km^−1^ travelled [51]. The estimated direct CO_2_ emissions or savings (in kg) during a bicycling/walking trip are therefore equal to the distance traveled in kilometer multiplied by 0.25 kg km^−1^. In energy expenditure, on average a person uses 35 Cal when traveling on a bicycle for a mile [51]. The calories burned during a bicycle trip are estimated as the distance traveled in km multiplied by 21.75 Cal km^−1^ (the metric equivalent). Similarly, energy expenditure on walking is based on bodyweight in pounds × 0.853 × distance in kilometers, assuming an average body weight of 150 lbs. The outputs also include estimated trip time, route length, total elevation gain, mean traffic pollution concentrations and degree of vegetation greenness of the chosen trip. Besides, an optimized route is linked to the slope gradient data and each roadway is color coded accordingly. Roadway by roadway directions and road length are displayed on the right side of the interface below the “Suggested Route”.

The associated software can be downloaded from https://drive.google.com/uc?export=download&id=0B9v9GRtvwvfoUDFPeGZ2SVNfUnc.

## Results and discussions

Using the popular and user-friendly Google Maps interface, the intervention tool allows a participant to locate fruit & vegetable stores, farmers’ markets, grocery stores, restaurants, recreational programs, licensed healthcare facilities and the number of traffic accidents within a specified buffer distance of the participant’s location of interest. In addition, a participant can also choose routes to and from amenities throughout the Los Angeles Metropolitan Area based on his/her preferences regarding safety, distance, elevation gain, air quality, and areas featuring trees and other vegetation. Users can choose trips that encourage physical activity such as by bicycling and walking or, if not, trips by car driving or through public transits. The trip planner calculates the number of calories burned over the course of a given route for bicycling and walking. It also estimates the emissions of CO_2_ from a car driving or the savings in CO_2_ emissions by bicycling and walking. A participant can visualize routes on the map interface through color-coded slope gradients. Other features such as geographic information of public parks, schools, educational institutions and surfaces of vegetation greenness and traffic-related air pollution are also available for assistance in route planning.

The usability and pilot testing of the online platform have been conducted in a related study [51]. This online tool is a tailored product from that bicycling trip planner for the purpose of overweight and obese intervention. The enhancement included helping participants identify healthy food stores at locations of their interest. It also included usage of recreational facilities to promote physical activity in those amenities. This tool also completed the previous bicycling trip planner by incorporating other modes of transportation including walking, public transportation and car driving. Because the related platform has gone through detailed user evaluation and pilot testing, we did not go through those tests again. Instead, this paper specifically focused on the functions of the tool for overweight and obese intervention, the required goal of the funded project.

The Google-based online intervention tool is part of the OPT Health II research project that targets families to effect the greatest possible change in the dietary behavior and physical activity of children and parents. Family environments are the key contents for the development of food preferences, patterns of food intake, and the development of activity preferences and patterns that shape children’s developing weight status [58]. Tailoring techniques are used to customize an intervention to individuals and their families based on their levels of behavior, perceptions, motivations, and attitudes for a particular target behavior. Tailored communications include printed materials such as newsletters, motivational interviewing and the online intervention tool. Printed materials have been effective on changing dietary outcomes, including food knowledge and choice [59], increasing fruit and vegetable consumption [60], and decreasing dietary fat consumption [61]. The OPT Health II uses tailored print communications to increase motivation, self-efficacy, and skills for dietary and physical activity behavior. Motivational interviewing is associated in adults with improved glycemic control [62], weight reduction [63], improvement in physical activity [64], increased fruit and vegetable consumption [65], and lower dietary fat [66]. Yet, few studies have applied motivational interviewing to diet and physical activity change in children or tested interventions in settings targeting families. The OPT Health II study uses motivational interviewing telephone calls to help families address personal, behavioral, and social concerns relating to diet/physical activity and obesity. Several studies have explored the utility of Geographic Information Sciences (GIS) in mapping disease and understanding disease etiology [67, 68]. Others have focused on GIS database processing capabilities for use in organizing health care systems [69, 70] and GIS network analysis techniques for assessing access to health facilities [71–73]. GIS has been used to characterize differential levels of accessibility enjoyed by population subgroups, to parks and open space, and other leisure resources linked to physical activity levels [74–78]. In Opt for Health II, we utilize the Google-based online intervention tool to provide tailored information for individuals and their families to identify locations of healthy food stores, recreational programs, and parks and green spaces surrounding homes of the families, and to provide families with optimized route options to reach amenities of interest. Similar to the bicycling trip planner for Metro Vancouver [51], this online intervention tool also promotes traveling by bicycling and by walking as forms of active transportation and help lower greenhouse gas CO_2_ and air pollutant emissions by reducing car trips. The online intervention tool services heightening the saliency of the newsletter materials [79–81].

Traditionally, obesity intervention programs have used a primary intervention approach and delivered within the healthcare setting [82–85]. This method makes delivery of intervention programs to large number of participants a daunting challenge [86]. Innovative approaches started to emerge which use static Internet contents (e.g., written materials published online) for interventions [86, 87]. The online tool developed here further enhances the obesity intervention strategy by adding interactive components to it: it helps participants locate healthy food stores and recreational programs and amenities for any neighborhoods in the Los Angeles Metropolitan Area and assist in finding corresponding optimized routes of travel. Traditional intervention strategies need to wait for a very lengthy time before they can be available for general public if such strategies are proven effective. This online tool, by contrast, can be released to general public for practice at any point. The overweight or obese people without participating in the experiment but with knowledge and choice of healthy foods and having interests in physical activity can use the tool directly to find their healthy foods and recreational programs and guide them to those amenities. For those people without enough knowledge and choice of healthy foods, this tool could guide them to those amenities and might lead to reduced non-healthy foods intake. In addition to serve people of overweight or obese, the system could also be used by general public as a tool for active transportation and help reduce CO_2_ emissions.

## Conclusions

We developed the first Google-based online intervention tool that assists obese and overweight participants in finding food and recreational amenities around a place and in locating optimized routes that fit their personal preferences. This tool overcomes obesity intervention programs that typically used a primary intervention approach and delivered within the health care setting. Because services in primary, secondary, and tertiary cares are often under-resourced, relatively uncoordinated with other parts of the health system, the effective management of intervention for a small number of participants is feasible; however, this management is ineffective for large number of participants. This online tool, by contrast, makes delivery of intervention programs to large number of participants feasible and can be released to general public for practice at any point. It is an important component in our continued effort on battling overweight and obesity.

Because the tool helps identify healthy food stores at locations of users’ interest, it can also serve general public for increasing healthy food shopping and eating options. The inclusion of parks, green spaces and recreational facilities can also help general public identify sites for physical activity and social gathering. The inclusion of air pollution surfaces helps susceptible population like asthma participants avoid locations of high air pollution, and the incorporation of traffic accidents data into the system raises safety awareness in every day travel. In addition, this tool can help promote bicycling and walking as forms of active transportation and help lower greenhouse gas carbon dioxide (CO_2_) and air pollutant emissions by reducing car trips. In summary, this tool can, in addition to serving overweight and obese population, also serve general public and policy makers for education, disease prevention and health promotion.

## Availability and requirements

Copyright © 2016 The Regents of the University of California (Regents). All Rights Reserved. Permission to use, copy, modify, and distribute this software and its documentation for educational, research, and not-forprofit purposes, without fee and without a signed licensing agreement, is hereby granted, provided that theabove copyright notice, this paragraph and the following two paragraphs appear in all copies, modifications, and distributions. Contact The Office of Technology Licensing, UC Berkeley, 2150 Shattuck Avenue, Suite 510, Berkeley, CA 94720-1620, (510) 643-7201, for commercial licensing opportunities.

Created by Jason G. Su, Department of Environmental Health Sciences, University of California, Berkeley, USA.

In no event shall Regents be liable to any party for direct, indirect, special, incidental, or consequential damages, including lost profits, arising out of the use of this software and its documentation, even if Regents has been advised of the possibility of such damage.

Regents specifically disclaims any warranties, including, but not limited to, the implied warranties of merchantability and fitness for a particular purpose. The software and accompanying documentation, if any, provided hereunder is provided "as is". Regents has no obligation to provide maintenance, support, updates enhancements or modifications.

## Abbreviations

U.S.A: United Sates of America
OPT: Obesity prevention tailored
BMI: Body mass index
ESRI: Environmental Systems Research Institute
AJAX: Asynchronous javascript and eXtensible markup language
SCAG: Southern California Association of Governments
MET: Metabolic equivalent
NDVI: Normalized difference vegetation greenness
PAR: Photosynthetically active radiation
SWITRS: Statewide integrated traffic records system
SPA: Shortest path analysis
CO_2_: Carbon dioxide
GIS: Geographic information sciences

## References

[1] Wang Y, Lobstein T (2006). Worldwide trends in childhood overweight and obesity. Int J Pediatr Obes.

[2] Ogden CL, Carroll MD, Curtin LR, McDowell MA, Tabak CJ, Flegal KM (2006). Prevalence of overweight and obesity in the United States, 1999–2004. JAMA.

[3] Overweight and Obesity: Data and Statistics. Accessed on 06/16/2015 from http://www.cdc.gov/obesity/data/facts.html.

[4] Jackson-Leach R, Lobstein T (2006). Estimated burden of paediatric obesity and co-morbidities in Europe. Part 1. The increase in the prevalence of child obesity in Europe is itself increasing. Int J Pediatr Obes.

[5] Lobstein T, Jackson-Leach R (2006). Estimated burden of paediatric obesity and co-morbidities in Europe. Part 2. Numbers of children with indicators of obesity-related disease. Int J Pediatr Obes.

[6] Lobstein T (2006). Commentary: obesity--public health crisis, moral panic or a human rights issue?. Int J Epidemiol.

[7] World Health Organization. Diet, nutrition and the prevention of chronic disease: report of a joint WHA/FAO expert consultation. WHO Tech Rep Ser. 2003; 916. 12768890

[8] Gill TP, Baur LA, King LA (2010). Should health policy focus on physical inactivity rather than obesity? No. BMJ.

[9] Oja P, Bull FC, Fogelholm M, Martin BW (2010). Physical activity recommendations for health: what should Europe do?. BMC Public Health.

[10] Fogelholm M (2010). Physical activity, fitness and fatness: relations to mortality, morbidity and disease risk factors. A systematic review. Obes Rev.

[11] CDC: How much physical activity do adults need? Accessed on 12/22/2015 through http://www.cdc.gov/physicalactivity/basics/ 2015.

[12] Oude Luttikhuis H, Baur L, Jansen H, Shrewsbury VA, O’Malley C, Stolk RP (2009). Interventions for treating obesity in children. Cochrane Database Syst Rev.

[13] Brown T, Avenell A, Edmunds LD, Moore H, Whittaker V, Avery L (2009). Systematic review of long-term lifestyle interventions to prevent weight gain and morbidity in adults. Obes Rev.

[14] Bruin JE, Saber N, Braun N, Fox JK, Mojibian M, Asadi A (2015). Treating Diet-Induced Diabetes and Obesity with Human Embryonic Stem Cell-Derived Pancreatic Progenitor Cells and Antidiabetic Drugs. Stem Cell Rep.

[15] Ochner CN, Tsai AG, Kushner RF, Wadden TA (2015). Treating obesity seriously: when recommendations for lifestyle change confront biological adaptations. Lancet Diabetes Endo.

[16] Scheen AJ, Paquot N (2015). A new paradigm for treating obesity and diabetes mellitus. Nat Rev Endocrinol.

[17] Al-Sabah SK, Almazeedi SM, Dashti SA, Al-Mulla AY, Ali DA, Jumaa TH (2015). The Efficacy of Laparoscopic Sleeve Gastrectomy in Treating Adolescent Obesity. Obes Surg.

[18] Ghai NR, Reynolds KD, Xiang AH, Massie K, Rosetti S, Blanco L (2014). Recruitment results among families contacted for an obesity prevention intervention: the Obesity Prevention Tailored for Health Study. Trials.

[19] Drewnowski A, Almiron-Roig E, Marmonier C, Lluch A (2004). Dietary energy density and body weight: is there a relationship?. Nutr Rev.

[20] Rolls BJ, Ello-Martin JA, Tohill BC (2004). What can intervention studies tell us about the relationship between fruit and vegetable consumption and weight management?. Nutr Rev.

[21] Hughes LA, Arts IC, Ambergen T, Brants HA, Dagnelie PC, Goldbohm RA (2008). Higher dietary flavone, flavonol, and catechin intakes are associated with less of an increase in BMI over time in women: a longitudinal analysis from the Netherlands Cohort Study. Am J Clin Nutr.

[22] Buijsse B, Feskens EJ, Schulze MB, Forouhi NG, Wareham NJ, Sharp S (2009). Fruit and vegetable intakes and subsequent changes in body weight in European populations: results from the project on Diet, Obesity, and Genes (DiOGenes). Am J Clin Nutr.

[23] Serdula MK, Byers T, Mokdad AH, Simoes E, Mendlein JM, Coates RJ (1996). The association between fruit and vegetable intake and chronic disease risk factors. Epidemiology.

[24] Glanz K, Yaroch AL (2004). Strategies for increasing fruit and vegetable intake in grocery stores and communities: policy, pricing, and environmental change. Prev Med.

[25] Parks and recreation programs declining as obesity, health concerns rise

[26] Fang CF, Ling DL (2003). Investigation of the noise reduction provided by tree belts. Landsc. Urban Plan..

[27] Yang J, McBride J, Zhou J, Sun Z (2005). The urban forest in Beijing and its role in air pollution reduction. Urban For Urban Green.

[28] Barnett TA, Lambert M, Kestens Y, Gauvin L, Van Hulst A, Daniel M (2009). Neighborhood Parks and Walking Among Youth at Risk of Obesity. Circulation.

[29] Wolch J, Jerrett M, Lam C, Chang CC, McConnell R, Reynolds K (2009). Childhood Obesity and Access to Public Recreational Programs. Obesity.

[30] Potestio ML, Patel AB, Powell CD, McNeil DA, Jacobson RD, McLaren L (2009). Is there an association between spatial access to parks/green space and childhood overweight/obesity in Calgary, Canada?. Int J Behav Nutr Phys Act.

[31] Wilks DC, Besson H, Lindroos AK, Ekelund U (2010). Objectively measured physical activity and obesity prevention in children, adolescents and adults: a systematic review of prospective studies. Obes Rev.

[32] Cliff DP, Okely AD, Morgan PJ, Jones RA, Steele JR (2010). The impact of child and adolescent obesity treatment interventions on physical activity: a systematic review. Obes Rev.

[33] Saquib N, Natarajan L, Rock CL, Flatt SW, Madlensky L, Kealey S (2008). The impact of a long-term reduction in dietary energy density on body weight within a randomized diet trial. Nutr Cancer.

[34] Pierce JP, Stefanick ML, Flatt SW, Natarajan L, Sternfeld B, Madlensky L (2007). Greater survival after breast cancer in physically active women with high vegetable-fruit intake regardless of obesity. J Clin Oncol.

[35] Wang D, Heckathorn SA, Mainali K, Hamilton EW (2008). Effects of N on plant response to heat-wave: a field study with prairie vegetation. J Integr Plant Biol.

[36] Pettorelli N, Vik JO, Mysterud A, Gaillard JM, Tucker CJ, Stenseth NC (2005). Using the satellite-derived NDVI to assess ecological responses to environmental change. Trends Ecol Evol.

[37] Pandit R, Laband DN (2010). Energy savings from tree shade. Ecol Econ.

[38] Kurn DM, Bretz SE, Huang H, Akbari H. The potential for reducing urban air temperatures and energy consumption through vegetative cooling. Berkeley Lab Heat Island Group. 1994; 1600.

[39] Jerrett M, Finkelstein MM, Brook JR, Arain MA, Kanaroglou P, Stieb DM (2009). A cohort study of traffic-related air pollution and mortality in Toronto, Ontario, Canada. Environ Health Perspect.

[40] Jerrett M, Buzzelli M, Burnett RT, DeLuca PF (2005). Particulate air pollution, social confounders, and mortality in small areas of an industrial city. Soc Sci Med.

[41] Krewski D, Jerrett M, Burnett RT, Ma R, Hughes E, Shi Y, et al. Extended follow-up and spatial analysis of the American Cancer Society study linking particulate air pollution and mortality. Res Rep Health Eff Inst. 2009;(140):5–114; discussion 115–136. http://www.ncbi.nlm.nih.gov/pubmed/19627030. 19627030

[42] Brunekreef B, Beelen R, Hoek G, Schouten L, Bausch-Goldbohm S, Fischer P (2009). Effects of long-term exposure to traffic-related air pollution on respiratory and cardiovascular mortality in the Netherlands: the NLCS-AIR study. Res Rep Health Eff Inst.

[43] Dubowsky SD, Suh H, Schwartz J, Coull BA, Gold DR (2006). Diabetes, obesity, and hypertension may enhance associations between air pollution and markers of systemic inflammation. Environ Health Perspect.

[44] Sun Q, Yue P, Deiuliis JA, Lumeng CN, Kampfrath T, Mikolaj MB (2009). Ambient air pollution exaggerates adipose inflammation and insulin resistance in a mouse model of diet-induced obesity. Circulation.

[45] Baja ES, Schwartz JD, Wellenius GA, Coull BA, Zanobetti A, Vokonas PS (2010). Traffic-related air pollution and QT interval: modification by diabetes, obesity, and oxidative stress gene polymorphisms in the normative aging study. Environ Health Perspect.

[46] Madrigano J, Baccarelli A, Wright RO, Suh H, Sparrow D, Vokonas PS (2010). Air pollution, obesity, genes and cellular adhesion molecules. Occup Environ Med.

[47] Su JG, Jerrett M, Beckerman B, Wilhelm M, Ghosh JK, Ritz B (2009). Predicting traffic-related air pollution in Los Angeles using a distance decay regression selection strategy. Environ Res.

[48] Su JG, Jerrett M, Beckerman B (2009). A distance-decay variable selection strategy for land use regression modeling of ambient air pollution exposures. Sci Total Environ.

[49] Winters M, Davidson G, Kao D, Teschke K (2011). Motivators and deterrents of bicycling: comparing influences on decisions to ride. Transp.

[50] Hochmair HH (2005). Towards a classification of route selection criteria for route planning tools. Developments in Spatial Data Handling.

[51] Su JG, Winters M, Nunes M, Brauer M (2010). Designing a route planner to facilitate and promote cycling in Metro Vancouver, Canada. Transp Res A Policy Pract.

[52] Dijkstra EW (1959). A note on two problems in connection with graphs. Numer Math.

[53] Dantzig GB (1960). On the shortest route through a network. Manag Sci.

[54] Floyd RW (1962). Algorithm 97: shortest path. Commun ACM.

[55] Current JR, ReVelle CS, Cohon JL (1984). The shortest covering path problem: an application of locational constraints to network design. J Reg Sci.

[56] Deo N, Pang C (1984). Shortest-path algorithms: taxonomy and annotation. Networks.

[57] Current JR, Pirkul H, Rolland E (1994). Efficient algorithms for solving the shortest covering path problem. Transp Sci.

[58] Birch LL, Davison KK (2001). Family environmental factors influencing the developing behavioral controls of food intake and childhood overweight. Pediatr Clin North Am.

[59] Kolodinsky J, Harvey-Berino JR, Berlin L, Johnson RK, Reynolds TW (2007). Knowledge of current dietary guidelines and food choice by college students: better eaters have higher knowledge of dietary guidance. J Am Diet Assoc.

[60] Pearson N, Atkin AJ, Biddle SJ, Gorely T (2010). A family-based intervention to increase fruit and vegetable consumption in adolescents: a pilot study. Public Health Nutr.

[61] Bourdeaudhuij I, Brug J (2000). Tailoring dietary feedback to reduce fat intake: an intervention at the family level. Health Educ Res.

[62] Martins RK, McNeil DW (2009). Review of Motivational Interviewing in promoting health behaviors. Clin Psychol Rev.

[63] Resnicow K, Davis R, Rollnick S (2006). Motivational interviewing for pediatric obesity: Conceptual issues and evidence review. J Am Diet Assoc.

[64] Miller ST, Marolen KN, Beech BM (2010). Perceptions of physical activity and motivational interviewing among rural African-American women with type 2 diabetes. Womens Health Issues.

[65] Campbell MK, Carr C, Devellis B, Switzer B, Biddle A, Amamoo MA (2009). A randomized trial of tailoring and motivational interviewing to promote fruit and vegetable consumption for cancer prevention and control. Ann Behav Med.

[66] Bowen D, Ehret C, Pedersen M, Snetselaar L, Johnson M, Tinker L (2002). Results of an adjunct dietary intervention program in the Women’s Health Initiative. J Am Diet Assoc.

[67] Cromley EK (2003). GIS and disease. Annu Rev Public Health.

[68] Gao S, Mioc D, Anton F, Yi X, Coleman DJ (2008). Online GIS services for mapping and sharing disease information. Int J Health Geogr.

[69] McLafferty SL (2003). GIS and health care. Annu Rev Public Health.

[70] Rushton G (2003). Public health, GIS, and spatial analystic tools. Annu Rev Public Health.

[71] Lovett A, Haynes R, Sunnenberg G, Gale S (2002). Car travel time and accessibility by bus to general practitioner services: a study using patient registers and GIS. Soc. Sci. Med..

[72] Freedman D, Perry G (2000). Body Composition and Health Status among Children and Adolescents. Prev Med.

[73] Parker EB, Campbell JL (1998). Measuring access to primary medical care: some examples of the use of geographical information systems. Health Place.

[74] Bock B, Marcus B, Pinto B, Forsyth L (2001). Maintenance of physical activity following an individual motivationally tailored intervention. Ann Behav Med.

[75] Nicholls S (2001). Measuring the accessibility and equity of public parks: A case study using GIS. Manag Leis.

[76] Talen E (1998). Visualizing fairness: Equity maps for planners. J Am Plann Assoc.

[77] Tarrant MA, Cordell HK (1999). Environmental justice and the spatial distribution of outdoor recreation sites: An application of geographic information systems. J Leis Res.

[78] Ryan C, Wilson J, Fulton W, Wolch J, Pastor M, Drier P (2002). Living on the edge: the region’s endangered species and habitats. Regional futures: public policy and making of 21st Century Los Angeles.

[79] French S, Story M, Jeffery R (2001). Environmental Influences on Eating and Physical activity. Annu Rev Pub Health.

[80] Saelens B, Sallis J, Frank L (2003). Environmental correlates of walking and cycling: findings from the transportation, urban design, and planning literatures. Ann Behav Med.

[81] Ricketts TC (2003). Geographic Information Systems And Public Health. Annu Rev Public Health.

[82] Rodriguez Cristobal JJ, Panisello Royo JM, Alonso-Villaverde Grote C, Perez Santos JM, Munoz Lloret A, Rodriguez Cortes F (2010). Group motivational intervention in overweight/obese patients in primary prevention of cardiovascular disease in the primary healthcare area. BMC Fam Pract.

[83] Beune EJ, Bindels PJ, Mohrs J, Stronks K, Haafkens JA (2010). Pilot study evaluating the effects of an intervention to enhance culturally appropriate hypertension education among healthcare providers in a primary care setting. Implement Sci.

[84] Hochhalter AK, Song J, Rush J, Sklar L, Stevens A (2010). Making the Most of Your Healthcare intervention for older adults with multiple chronic illnesses. Patient Educ Couns.

[85] McNamara R, Robling M, Hood K, Bennert K, Channon S, Cohen D (2010). Development and Evaluation of a Psychosocial Intervention for Children and Teenagers Experiencing Diabetes (DEPICTED): a protocol for a cluster randomised controlled trial of the effectiveness of a communication skills training programme for healthcare professionals working with young people with type 1 diabetes. BMC Health Serv Res.

[86] Gabriele JM, Stewart TM, Sample A, Davis AB, Allen R, Martin CK (2010). Development of an internet-based obesity prevention program for children. J Diabetes Sci Technol.

[87] McDoniel SO, Wolskee P, Shen J (2010). Treating obesity with a novel hand-held device, computer software program, and Internet technology in primary care: the SMART motivational trial. Patient Educ Couns.

